# Identification of Secondary Metabolites by UHPLC-ESI-HRMS/MS in Antifungal Strain *Trichoderma harzianum* (LBAT-53)

**DOI:** 10.3390/jof10080547

**Published:** 2024-08-03

**Authors:** Giselle Hernández, Amaia Ponce de la Cal, Yuset Louis, Yamilé Baró Robaina, Yamilet Coll, Iraida Spengler, Yaneris Mirabal-Gallardo

**Affiliations:** 1 Center for Natural Products Research, Faculty of Chemistry, University of Havana, Havana 10400, Cuba; 2 Plant Health Research Institute (INISAV), 110 Str. 514, Havana 11600, Cubarobainabaro@gmail.com (Y.B.R.); 3 Faculty of Engineering, Institute of Applied Chemistry, Universidad Autónoma de Chile, Talca 3460000, Chile

**Keywords:** natural products, UHPLC-ESI-MS/MS, metabolites, fungi, biocontrol, phytopathogens

## Abstract

*Trichoderma* spp. are filamentous fungi generally observed in nature, which are widely marketed as biocontrol agents. The secondary metabolites produced have obtained special attention since they possess attractive chemical structures with a broad spectrum of biological activities. In Cuba, the species of *Trichoderma* have been commercially applied for the control of several phytopathogens to protect agricultural crops, but few studies have been carried out to detect and characterize the production of metabolites with biological activity. The strain *Trichoderma harzianum* LBAT-53 was subjected to an antifungal in vitro assay against *Fusarium oxysporum* f.sp. *cubense* by dual culture and volatile metabolite assays and fermented in PDB under constant agitation conditions. The ethyl acetate crude extract was obtained by liquid–liquid extraction. The fungal extract was investigated for the composition of secondary metabolites through chemical screening and ultrahigh performance liquid chromatography-tandem mass spectrometry (UHPLC-MS/MS) in negative ionization mode. As a result, LBAT-53 showed antagonistic activity in vitro (Class 2) against the pathogen evaluated in direct confrontation (76.9% of inhibition in 10 days) and by volatile metabolites (<40% in 7 days). Furthermore, seven low-molecular-weight phenolic compounds, including chrysophanol, phomarin, endocrocin, and trichophenol A, among others, were identified using UHPLC-ESI-MS/MS. This study is the first work on the characterization of secondary metabolites produced by the commercially applied strain LBAT-53, which is a promising source of bioactive compounds. These results provide a better understanding of the metabolism of this fungus, which is widely used in Cuba as biopesticides in agriculture pest control.

## 1. Introduction

As the global population continues to grow, food demand increases and production needs to increase [1]. Several factors have a negative impact on crop production, including pathogens that affect the quality and yield of crops. Fusarium Wilt of Banana, caused by the fungus *Fusarium oxysporum* f.sp. *cubense* Schltdl. (Foc), is considered one of the most destructive vascular wilt fungal diseases recorded in banana history. Throughout the world, the yearly losses of banana owing to this disease range from 60% to 90% [2,3]. It is a limiting factor in global production, and the fight against it represents one of the highest production costs [4]. In Cuba, the spread of Fusarium Wilt has caused a marked impact on production costs and especially on the clonal structure of the planted surface [2].

Humans use chemical control mechanisms that affect ecosystems to combat pests and diseases; however, increased resistance of pests to these chemically synthesized products has been observed [5]. In this scenario, the scientific community has focused its research on the use of alternatives that are more environmentally friendly and safer for producers and consumers. The use of biological control agents is a new alternative to fight fungal diseases [6] and has gained great interest in the last years in many pathosystems, including Foc/banana. This has been mainly due to the large input of pesticides, which cause economic, environmental, and safety concerns. Greenhouse and in vitro studies have reported microorganisms that are antagonistic to Foc [7].

*Trichoderma* spp. are distributed widely in soil and have been developed as a source of biocontrol agents for years [8,9]. These fungi are remarkable for their rapid growth and utilization of diverse substrates. In addition, they use different mechanisms, including antibiosis, mycoparasitism, pathogen competition, plant growth promotion, resistance to biotic and abiotic stresses, and activation of a pathogen defensive system [6,10,11,12]. Their use as a biocontrol agent against phytopathogens that cause losses in important agricultural crops such as *Sclerotium rolfsii*, *Macrophomina phaseolina*, *Botrytis cinerea*, *Rhizoctonia* spp. and *Fusarium* spp. is highlighted [13,14,15,16], and even with oomycetes such as *Pythium ultimum* [17].

*Trichoderma* spp. have the ability to produce different kinds of chemical substances, like volatile and nonvolatile compounds, polyketides, and siderophores, that can promote biocontrol activities [18]. A wide variety of compounds have been identified during the interaction between *T. harzianum* and *Rhizoctonia solani*, such as heptelidic acid, trichoviridine, harzianic acid, gliotoxin, glioviridin, viridin, and viridiol. The ability of the same strain of *Trichoderma* to secrete several antifungal compounds simultaneously limits the risk of the appearance of microorganisms resistant to these metabolites, a relevant aspect from a practical point of view [19].

Analytical methods including spectrophotometry, high performance liquid chromatography (HPLC), gas chromatography-tandem mass spectrometry (GC-MS/MS), high pressure liquid chromatography mass spectrometry (LC-MS), and, most recently, ultrahigh performance liquid chromatography-tandem mass spectrometry (UHPLC-MS/MS), to facilitate rapid and accurate identification of chemical compounds in complex mixtures, refs. [12,20,21,22,23] have been development for several years for the identification of secondary metabolites.

In Cuba, species of *Trichoderma* have also been used for several years to improve crop yields; specifically, the strains *Trichoderma harzianum* Rifai LBAT-34 and LBAT-53, as well as *Trichoderma viride* Persoon LBAT-TS3, are mass-produced in the Centers for the Reproduction of Entomophages and Entomopathogens (CREE) on solid support and also on liquid and agitated cultures [24]. Currently, these Cuban strains of *Trichoderma* are used within the national Integrated Pest Management (IPM) programs in crops of economic importance and have been widely used and commercially applied for the control of phytopathogenic fungi and nematodes as antagonists in soils to protect different types of crops, such as beans and bananas [25,26,27,28,29].

The production of *Trichoderma* for its application in the control of soil phytopathogens is an important line of research in the Plant Health Research Institute (INISAV). Specifically, the *T. harzianum* strain LBAT-53 is the active ingredient of the commercial product TRICOSAVE 53. For a further evaluation of its biological potential, the study of its antagonism against Foc is required as a pathogen of great importance for the country. Furthermore, the secondary metabolites produced by this strain, which are considered to play a significant and effective role in suppressing plant pathogens and promoting growth, has not yet been characterized.

The aim of this research was to identify the secondary metabolites present in the crude extract of ethyl acetate through chemical screening and UHPLC-MS/MS and evaluate the antifungal activity in vitro of *Trichoderma harzianum* LBAT-53 against *Fusarium oxysporum* f.sp. *cubense*, the causal agent of Fusarium Wilt, also known as Panama disease, a severe fungal disease in banana.

## 2. Materials and Methods

### 2.1. Fungal Strains

The *Trichoderma harzianum* strain LBAT-53 from INISAV Microbial Culture Collection (Havana, Cuba) was used in this study.

The phytopathogen *Fusarium oxysporum* f.sp. *cubense* (Foc) PalPR7 race 1 was collected from the municipality of Los Palacios, Pinar del Río, Cuba, and is deposited in the Microbial Culture Collection of INISAV.

All the fungal strains were cultured on potato dextrose agar (PDA) plates for 5 days in darkness at 30 ± 1 °C.

### 2.2. Fermentation Process

To obtain liquid cultures, the strain LBAT-53 was inoculated in 2 L Erlenmeyer flasks with 1 L of sterile potato dextrose broth (PDB). The inoculation was carried out from 5 mm diameter plugs obtained from actively growing margins of PDA cultures. The cultures were placed under constant agitation, 150 rpm for 7 days at 30 ± 1 °C. They were subsequently filtered under vacuum through filter paper (Whatman No. 4; Brentford, UK) to eliminate the mycelium and obtain the supernatant, which were centrifuged at 8000 rpm and filtered again on a 0.2 µm nitrocellulose membrane.

### 2.3. Antifungal In Vitro Assay

The antifungal activity of *T. harzianum* LBAT-53 against *F. oxysporum* f.sp. *cubense* was performed by a dual culture technique and volatile metabolite assay.

First, culture disks from a 7-day-old culture dish of both fungi (5 mm diameter from growing edge colonies) were placed on the opposite ends of Petri dishes with PDA (90 mm) and incubated at 30 °C in the dark for 10 days. As control, a disk of mycelium of the phytopathogen was placed on separate plates. The 5-grade Class Scale described by Bell et al. [30] was used to classify the *Trichoderma* strain.

The evaluation of volatile metabolites was carried out according to the methodology described by Dennis and Webster [31]. Culture disks of both fungi (5 mm) were inoculated in the center of Petri dishes with PDA (90 mm), and the lids were removed. The bottoms containing an antagonist and a pathogen were placed together and sealed using Parafilm^®^ M (Darmstadt, Germany)and incubated at 30 ± 1 °C under dark conditions. The pathogen was in the upper plate in order to avoid any interference by antagonistic spores in the plate inoculated with Foc. As control, a bottom containing the pathogen was used, overlapping with another containing only PDA medium (Section S1).

All experiments were performed in triplicate. The radial growth of the pathogen was measured every 24 h with a graduated ruler, and the percentage of radial growth inhibition (PRGI) was calculated according to Hernández et al. [32].

### 2.4. Extraction of Secondary Metabolites

The fermentation broth of the *Trichoderma* strain LBAT-53 was filtered with a Whatman filter paper No. 2 (Brentford, UK) by gravity to separate the insoluble components present in the fermentation broth. The pH of the supernatants was adjusted to 2.0 with H_3_PO_4_ and then extracted with ethyl acetate (3 × 25 mL), concentrated in a rotary evaporator (Büchi R-300, BÜCHI Labortechnik GmbH, Essen, Germany) coupled to a vacuum pump (Büchi V-300, BÜCHI Labortechnik GmbH, Essen, Germany) to yield the crude metabolite, which was re-suspended in 2 mL of methanol, and 10 μL was injected for each analysis.

### 2.5. Chemical Screening

The phytochemical screening for the ethyl acetate crude extract of LBAT-53 was analyzed after extraction with ethanol. The different chemical groups were characterized with reference to the Rondina and Coussio [33] technique, with slight modifications (Section S2).

### 2.6. Cleanup of the Sample

An amount of 2 mL of MeOH/H_2_O 8:2 (*v*/*v*) solution of the extract (1 mg/mL) was subjected to solid-phase extraction using SPE cartridges Chromabond^®^ C18 (loading, 200 mg/3 mL; particle size, 54 μm, Macherey-Nagel, Düren, Germany) eluted with MeOH/H_2_O 8:2 (*v*/*v*). After drying, 1 mg was dissolved in 1 mL of MeOH/H_2_O 8:2 (*v*/*v*) solution (solution A), and an aliquot (10 μL) was diluted with MeOH/H_2_O 8:2 (*v*/*v*) up to a final volume of 1 mL, and was filtered through a 0.22 μm membrane of a nylon filter. The solution (150 μL) was diluted with MeOH/H_2_O 8:2 (*v*/*v*) up to a final concentration of 1 ppm and submitted to UHPLC-ESI-HRMS/MS.

### 2.7. UHPLC-ESI-HRMS/MS Conditions and Data Analysis

The negative ion high-resolution ESI mass spectra were obtained from an Orbitrap Elite mass spectrometer (Thermo Fisher Scientific, Bremen, Germany) equipped with a heated ESI electrospray ion source (spray voltage negative ion mode, 3.5 kV; source heater temperature, 150 °C; capillary temperature, 325 °C; FTMS resolution, 30,000). Nitrogen was used as sheath and auxiliary gas. The MS system was coupled online to an ultrahigh performance liquid chromatography (UHPLC) system (Dionex UltiMate 3000, Thermo Fisher Scientific), equipped with a RP C18 column (1.8 µm; 100 × 1.0 mm; ACQUITY UPLC HSS T3 C18; Waters, column temperature, 45 °C), and a photodiode array detector (PDA, Thermo Fisher Scientific). For the UHPLC, a gradient system was used starting from H_2_O (A; Milli-Q, Merck Millipore, Burlington, MA, USA):CH_3_CN (B; Chromasolv LC-MS, Riedel-de Haen, Honeywell, Charlotte, NC, USA) 95:5 (each of them containing 0.1% (*v*/*v*) formic acid (eluent additive for LC-MS, Honeywell Fluka, Charlotte, NC, USA), isocratic for 1 min) raised to 50:50 within 3 min and in further 10 min to 10:90 to finally 5:95 within 1 min to then hold on 5:95 for further 3 min, the flow rate at 150 µL min^−1^. The wavelength range of the PDA measurements was 190–400 nm used for detection. The CID tandem mass spectra (buffer gas: helium; FTMS resolution, 15,000) were recorded in data-dependent acquisition mode (DDA) using normalized collision energies (NCE) of 35%. The instrument was externally calibrated by the Pierce^®^ LTQ Velos ESI negative ion calibration solution (product number 88324, Thermo Fisher Scientific, Rockford, IL, 61105 USA). The data were evaluated by the Xcalibur software 2.2 SP1 (Thermo Fisher Scientific).

### 2.8. Statistical Analysis

The experimental design was completely random, and the data obtained were processed using a simple classification Analysis of Variance (ANOVA) with its corresponding significance test (*p* ˂ 0.05) according to Tukey’s test using the statistical software InfoStat version 2008 for Windows.

## 3. Results

### 3.1. Antifungal In Vitro Assay

**Figure 1:**
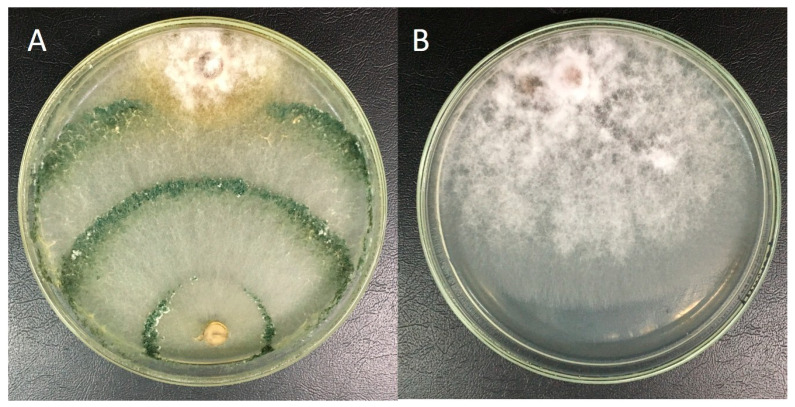
Dual culture of the strain *T. harzianum* LBAT-53 and *F. oxysporum* f.sp. *cubense* PALPR7 after 10 days. (**A**) Dual culture. (**B**) Control of *F. oxysporum* f.sp. *cubense* PALPR7.

The results obtained in dual culture showed that the *T. harzianum* LBAT-53 strain has a higher growth rate than *F. oxysporum* f.sp. *cubense* PalPR7. After 10 days, it was placed in Class 2 on the Bell et al. scale [30], showing a high antagonistic effect, with the growth of the pathogen stopping upon contact with the antagonist mycelium (Figure 1).

In the interaction with the pathogenic strain PalPR7 at 48 h, prior to physical contact, a growth inhibitory effect greater than 10% was observed due to the effect of the LBAT-53 strain, with significant differences with the control treatment (Table 1). At 7 days, the inhibitory effect was greater than 66%, with significant differences compared with the control, and after 10 days, the percentage of inhibition of pathogen growth was 76.90%.

**Figure 2:**
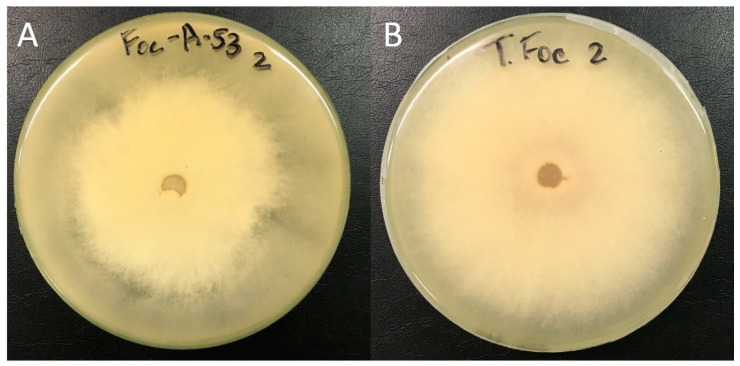
Effect of volatile metabolites of *Trichoderma harzianum* LBAT-53 on *F. oxysporum* f.sp. *cubense* PalPR7. (**A**) Confrontation. (**B**) Control of *F. oxysporum* f.sp. *cubense* PalPR7.

Regarding the release of volatile metabolites, the strain LBAT-53 inhibited the growth of *F. oxysporum* f.sp. *cubense* PalPR7. At 72 h, the percentage of inhibition in the mycelium of *F. oxysporum* f.sp. *cubense* presented significant differences with the control. After 7 days, growth inhibition was greater than 40% due to the release of volatile metabolites (Table 1), inducing changes in the coloration and shape of the pathogen colony (Figure 2).

### 3.2. Chemical Screening

The chemical screening of a crude extract of *T. harzianum* LBAT-53 revealed the presence of amine compounds, saponins, and glycosylated triterpenes–steroids; moderate amounts of free flavonoids and quinones; and higher amounts of phenols and reducing sugars. On the other hand, no free triterpenes–steroids, alkaloids, cardenolides, glycosylated flavonoids, and proanthocyanidins/catechins were detected (Table 2). A detailed description of the procedure to obtain each fraction of increasing polarity and the assays corresponding to the different groups of metabolites detected is given in the Supplementary Information (Section S2).

### 3.3. UHPLC-ESI-MS/MS

**Figure 3:**
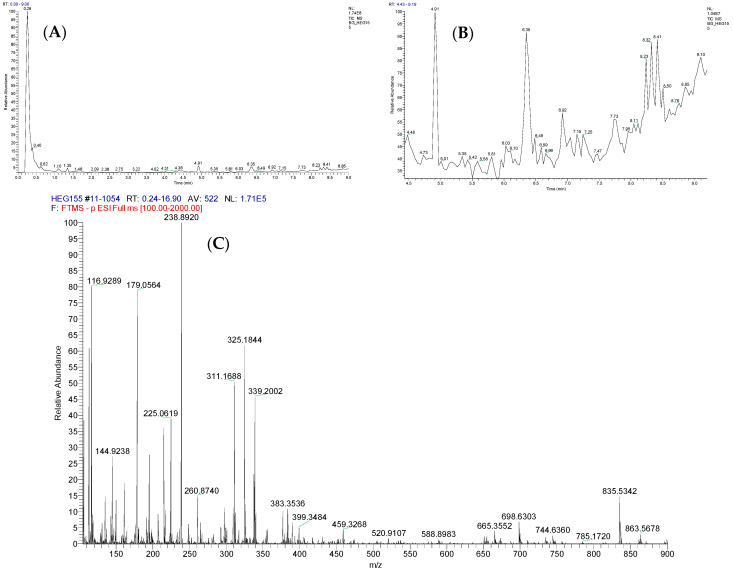
(**A**) Total ion chromatogram (TIC) obtained for ethyl acetate from *T. harzianum* LBAT-53 by reversed-phase UHPLC-ESI-MS/MS, (**B**) expanded ion chromatogram of metabolites with a Rt = 4–9.5 min, and (**C**) total ion spectrum of the ethyl acetate extract obtained from the culture broth of the strain LBAT-53 in negative ionization mode.

The extract of *T. harzianum* was also analyzed using UHPLC-ESI-MS/MS in negative ion mode. Figure 3A corresponds to the chromatogram of the total ions obtained in a UHPLC equipped with a Watters RP C18 column (1.8 µm; 100 × 1.0 mm), and Figure 3B to the expanded chromatogram of the metabolites eluted with a Rt of 4–9.5 min, where, considering the characteristics of the reversed phase, in this region, the metabolites of medium polarity should appear. The spectrum of total ions in the extended *m*/*z* 140–900 region afforded characteristic deprotonated ions corresponding to the different metabolites present in the crude extract (Figure 3C).

#### 3.3.1. Anthraquinone Identification

**Figure 4:**
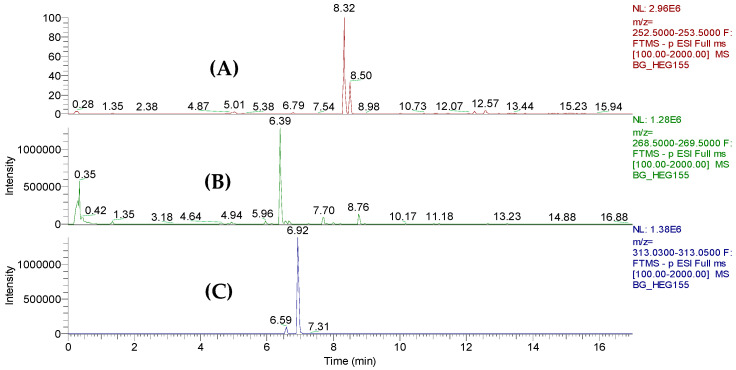
Extracted ion chromatograms (**A**–**C**) obtained from the ethyl acetate crude extract of *T. harzianum* (LBAT-53) based on the theoretical masses of anthraquinones studied by Laub et al. [34].

**Figure 5:**
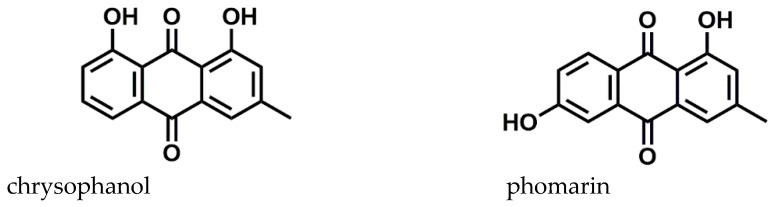
Structures of anthraquinones chrysophanol and phomarin identified in ethyl acetate crude extract of *T. harzianum* LBAT-53 by UHPLC-ESI-MS/MS in negative ion mode.

The presences of the different anthraquinones within the extract are visualized in the extracted ion chromatograms (EICs) at different retention times (Figure 4A–C). The assignment of the structures is based on their elemental composition determined by high-resolution mass spectrometry (Table 3). For the data evaluation, the target *m*/*z* values were extracted from the total ion chromatogram to obtain the corresponding extracted ion chromatograms for each compound. Due to the resolving power of the Orbitrap detector, a differentiation of isobaric ions was possible, as shown in the EIC of Figure 4A, where the anthraquinone peak at *m*/*z* 253.0515 is clearly separated from other accompanying ion at the same nominal mass. In the second-order spectrum of the pseudomolecular ion with Rt = 8.32 min, common losses of H_2_O and CO were observed. The compound was identified as chrysophanol according to the fragmentation pattern, and the second peak, eluting at 8.50 min, was tentatively identified as phomarin (1,6-dihydroxy-3-methyl-9,10-anthracenedione), a positional isomer of chrysophanol (Figure 5).

**Figure 6:**
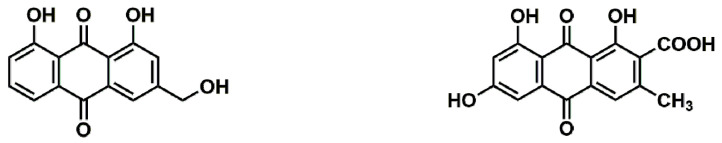
Structures of anthraquinones detected.

Two additional anthraquinones—1,8-dihydroxy-3-(hydroxymethyl)anthracene-9,10-dione (*m*/*z* = 269.0457 with Rt = 6.49 min and endocrocin (*m*/*z* = 313.0353 with Rt = 6.95 min)—were identified (Figure 6) based on combined data available on the mass spectra with molecular weight and characteristic fragment ions that were previously identified by Laub et al., 2020 [34].

#### 3.3.2. Other Phenolic Compounds

**Figure 7:**
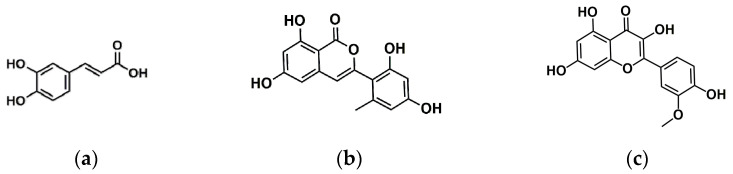
Caffeic acid (**a**), trichophenol A, (**b**) and isorhamnetin (**c**) detected in the crude extract of ethyl acetate from *T. harzianum* (LBAT-53).

Caffeic acid (*m*/*z* 179.0564), trichophenol A (*m*/*z* 299.2593), and isorhamnetin (*m*/*z* 315.0545) were identified on the ethyl acetate crude extract of *T. harzianum* (LBAT-53). The structures of each compound are shown in Figure 7.

## 4. Discussion

*Trichoderma* species are the most studied and used fungi for the control of plant disease [35]. Diverse studies revealed the different mechanisms that *Trichoderma* has as a biocontrol agent [8,9,27,36,37,38,39]. In 2016, Khaledi and Taheri investigated the biocontrol mechanisms of 11 *Trichoderma* isolates against *M. phaseolina* in dual culture tests [40]. The results showed that all isolates inhibited the mycelial growth of the pathogen from 20.2 to 58.7%. Likewise, in Cuba, in a study conducted by Martínez-Coca et al. (2018) [41], most of the *Trichoderma* strains evaluated inhibited the growth of *Fusarium* spp. isolates by over 40%. When specifically evaluating the LBAT-53 strain, Sierra et al. (2007) obtained the highest inhibition percentages for the pathogens *Fusarium subglutinans* and *Rhizoctonia solani*, with values of 48% and 58%, respectively [42]. The PRGI reported in this study are higher than the values reported by these authors.

In this study, *T. harzianum* (LBAT-53) displayed a faster growth compared with *F. oxysporum* and inhibited the mycelial growth of the pathogen in more than 66% at 7 days. In dual culture assays with Foc race 1 strains, several authors have obtained inhibition values of 38.82% and 53.46% with the *Trichoderma* sp. TB1 isolate [43]. Hernández-Melchor et al. [44] evaluated 15 native *Trichoderma* isolates against 5 *Fusarium oxysporum* f.sp. *cubense* RACE 1 isolates, and the best 8 strains showed inhibition percentages in the range of 3% to 54%; the values were lower than those obtained in this study. These results could be explained because of the highest competition for nutrients and space of LBAT-53. Furthermore, the excretion of metabolites with significant fungistatic action over the pathogen was also corroborated with inhibition values greater than 40%. Recently, Lakhdari et al. [45] corroborated the presence of volatile metabolites with antifungal activity present in the ethyl acetate and *n*-butanol extracts of *T. harzianum*.

Secondary metabolites in nature perform specialized functions at very low concentrations. They facilitate symbiosis with microorganisms, insects, plants, and higher animals, and are considered mediators of chemical communication between soil inhabitants in different ecological niches [8,37]. Since they are produced in very low concentrations, analytical methods for detecting analytes present at trace levels in a complex matrix and screening large numbers of samples have been developed. Among others, ultrahigh performance liquid chromatography-tandem mass spectrometry (UHPLC-MS/MS) is a better choice due to higher sensitivity, higher resolution, and a shorter run-time. However, conventional chemical assays remain the choice for preliminary chemical screening because they are inexpensive and simple and require fewer resources [46]. These techniques combine metabolite solubility with solvent polarity and pH variation during the extraction procedure. The crude extract is separated into fractions that are subjected to qualitative assays of color development and/or solid precipitation, depending on the type of metabolite.

In this study, the chemical screening allowed for detecting the presence of phenolic compounds. These kinds of secondary metabolites were identified in different species of *Trichoderma* (*T. polypore*, *T. polyalthiae*, and *T. gamsii*) [47]. In the medium-high polarity fraction corresponding to the CHCl_3_/EtOH extract (Fraction D), the Shinoda assay was positive, suggesting a moderate presence of flavonoids such as flavonones, flavones, flavanonols, chalcones, and flavonols. Ni et al. [48] identified flavonoids, for example, dihydromyricetin, isorhamnetin, and 4-hydroxy-5,7-dimethoxyflavanone, present in the fermentation broth of *T. asperellum* TJ01. Ninhidrine addition to the respective fraction A and an aqueous fraction F produced a slightly violet color, indicating the presence of amine compounds. The Börntrager assay for quinones detection was positive, and the color of a solution indicated the presence of anthraquinones. This family of compounds was also detected by Dennis et al. [31]. They identified chrysophanol, phomarin, emodin, and other anthraquinones from the *T. harzianum* strain Th-R16 [49]. In the scientific literature consulted, it was found that several investigations have shown that *Trichoderma* species are capable of producing these metabolites [50]. Anthraquinones are very important for their antifungal, antimicrobial, and antioxidant action, among others [50,51,52]. They have been considered among the most abundant fungal natural products [53]. Based on results of chemical screening, the strain LBAT-53 was characterized for the first time with a highly sensitive method like UPLC-ESI-MS/MS in negative ionization mode. Four anthraquinones were detected and corroborated their structures according to the fragmentation patterns observed. In the case of chrysophanol, the antifungal activity that this molecule presents against *Blumeria graminis* f.sp. *Hordei*, *Podosphaera xanthii*, *Candida albicans*, *Cryptococcus neoformans*, *Trichophyton mentagrophytes*, and *Aspergillus fumigatus* is well documented [54]. When tested against *Botrytis cinerea* and *Rhizoctonia solani*, it also showed inhibition of the growth of these pathogens [55]. Liu et al. [55] demonstrated that chrysophanol is involved in the stimulation of plant growth, photosynthesis, and the induction of host defense responses during *T. harzianum* colonization on cabbage. The anthraquinone phomarin, which is a positional isomer of chrysophanol, detected in the crude acetate extract of the Cuban strain LBAT-53, was also identified in *Trichoderma* species [56].

**Figure 8:**
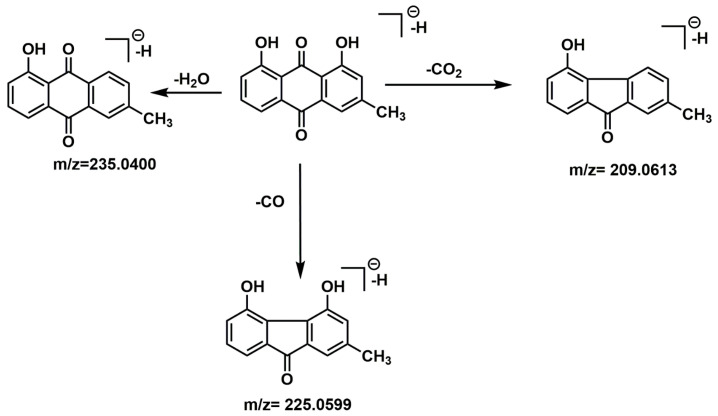
Fragmentation pathway proposed for chrysophanol, [M−H]^−^ ion at *m*/*z* 253 according to the MS/MS spectra.

**Figure 9:**
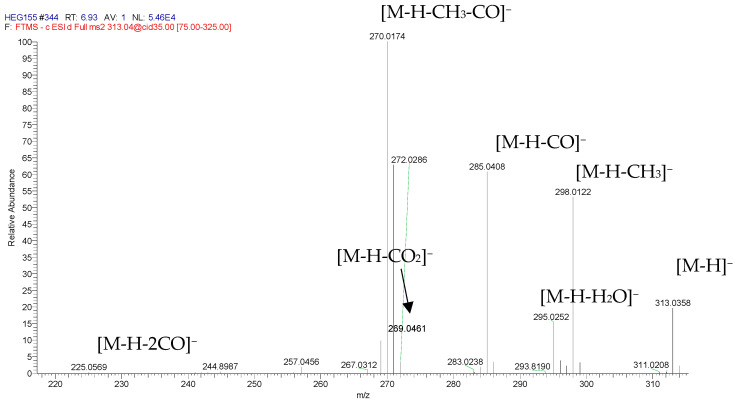
Second-order spectra of endocrocin.

As an example, the fragmentation patterns of chrysophanol and endocrocin were examined by Ms^2^ analysis (Figure 8 and Figure 9). Characteristic product ions of chrysophanol corresponded to loss of a neutral molecular fragment, such as CO_2_ resulting in a product ion at *m*/*z* = 209, H_2_O [M-H-18]^−^ at *m*/*z* = 235, and CO resulting in the base peak at *m*/*z* = 225 was detected. In Figure 8 is shown the fragmentation pathway proposed for this molecule.

Characteristic losses in the second-order spectra of endocrocin were detected under negative ion electrospray (Figure 9). The loss of a neutral molecular fragment was also a characteristic of methylated anthraquinones [57]. The main product ion corresponds to [M-H-CH_3_-CO]^−^ (base peak) obtained from ion at *m*/*z* = 298 [M-H-CH_3_]^−^, which corresponds to the loss of the methyl group; this ion is further decomposed by the loss of CO.

The isocoumarin trichophenol A, detected in LBAT-53, was isolated and reported by Liu et al. in 2020 from the marine-alga-endophytic fungus *Trichoderma citrinoviride* A-WH-20-3 [58]. The flavonol isorhamnetin also was identified in this study. In a research conducted by Unver [59] in 2024, the antifungal activity was demonstrated with a significant inhibitory effect (MIC = 1.875 mg/mL) against *Candida* species. This bioactive metabolite was reported by Ni et al. [48] recently by liquid chromatography coupled to mass spectrometry with a triple quadrupole analyzer. The authors studied the metabolic changes in *Trichoderma asperellum* TJ01 at different fermentation times. Additionally, they detected at 72 h that the culture had a higher proportion of upregulated flavonoids.

In a study conducted by Zakaba and Pavela in 2013 [60], the authors tested 21 phenolic compounds against filamentous fungi and demonstrated that caffeic acid had an inhibitory effect on the mycelial growth of *F. oxysporum* at a basic concentration of 1000 μg/mL with 33.33 ± 0.09% of inhibition.

In general, the secondary metabolites of *Trichoderma* confer the biocontrol activity of the strain either by directly inhibiting pathogens (direct antagonism) of the host or by inducing host plant resistance [49]. They are essential for fungal development and actively determine interactions with other organisms. Compounds possessing a phenolic ring system have been found to exhibit a number of pharmacological properties; for example, some of the phenolic compounds, such as phenolic acids, flavonoids, catechins, anthocyanins, tannins, anthraquinones, and naphthoquinones, which are lipophilic in nature, can inhibit the activity of the ABC transporters [61]. Polyphenols can also bind directly to proteins, interfering with the tertiary structure of proteins and thus effectively inhibiting the function of ABC transporters, which could affect the pathogenicity mechanisms of the fungus that causes the disease in the plant. The structure of phenolic compounds is such that they can diffuse through the microbial membranes and can penetrate into the cell, where they can interfere in the metabolic pathways [61]. According to these results, the anthraquinones detected in this research could be involved directly in the biocontrol function of *T. harzianum* against the pathogen tested, and a synergistic effect with other phenolic compounds identified also could be observed.

## 5. Conclusions

This investigation represents a first approach to the chemical study of the *Trichoderma harzianum* (LBAT-53) strain from the Microbial Culture Collection of INISAV. Chemical screening and a highly sensitive and high-resolution UHPLC-ESI-MS/MS technique were used as qualitative methods to identify secondary metabolites produced by this important strain, which is used as a biocontrol agent. In vitro antifungal activity showed that LBAT-53 has an antagonistic effect against *Fusarium oxysporum* f.sp. *cubense* PalPR7. The results support the potential use of *Trichoderma harzianum* as an alternative for the control of this pathogen, which affects plantains and banana crops worldwide.

## Figures and Tables

**Table 1:** Effect of *T. harzianum* LBAT-53 on the radial growth of *F. oxysporum* f.sp. *cubense* PalPR7.

Treatment	Percentage of Radial Growth Inhibition(%)									
	Dual Culture					Volatile Metabolites				
	24 h	48 h	72 h	7 d	10 d	24 h	48 h	72 h	7 d	8 d
Control PalPR7	0 ^a^	0 ^a^	0 ^a^	0 ^a^	0 ^a^	0 ^a^	0 ^a^	0 ^a^	0 ^a^	0 ^a^
LBAT-53 vs. PalPR7	16.67 ^a^	10.17 ^b^	14.94 ^ab^	66.09 ^cd^	76.90 ^cd^	20.83 ^ab^	8.53 ^abc^	14.14 ^cd^	40.65 ^cd^	44.44 ^e^

Means with different letters, in the same column, indicate significant differences (ANOVA and Tukey test, *p* ≤ 0.05).

**Table 2:** Chemical screening of tested extract from *Trichoderma harzianum* LBAT-53.

Family Compounds	LBAT-53
**Fraction A**	
Phenols	+++
Amine compounds	+
**Fraction B**	
Triterpenes/steroids	-
Quinones	++
**Fraction C1**	
Alkaloids	-
**Fraction C2**	
Triterpenes/steroids	-
Alkaloids	-
Cardenolides	-
**Fraction D**	
Flavonoids	++
Cardenolides	-
Alkaloids	-
Proanthocyanidins/catechins	-
Triterpenes/steroids	+
**Fraction E**	
Proanthocyanidins/catechins	-
Flavonoids	-
Reducing sugars	+++
**Fraction F**	
Saponins	+
Amine compounds	+++

**Legend:** (-): Negative test (absence of turbidity or precipitation). (+): Weak positive test. (++): Positive test (moderate amount). (+++): Test strongly positive.

**Table 3:** Anthraquinones: elemental composition and exact mass.

Ionic Species	Rt (min)	Elemental Composition	[M−H]^−^ (*m*/*z* Theoretical)	[M−H]^−^ (*m*/*z* Experimental)	RDB	Δ ppm
**[M−H]^−^**	8.32	C_15_H_9_O_4_^−^	253.0579	253.0515	11.5	0.66
**[M−H]^−^**	8.50	C_15_H_9_O_4_^−^	253.0579	253.0515	11.5	0.66
**[M−H]^−^**	6.39	C_15_H_9_O_5_^−^	269.0455	269.0459	11.5	0.57
**[M−H]^−^**	6.92	C_16_H_9_O_7_^−^	313.0354	313.0350	12.5	−1.09

## Data Availability

The original contributions presented in the study are included in the article and Supplementary Materials, further inquiries can be directed to the corresponding authors.

## Supplementary Materials

The following supporting information can be downloaded at https://www.mdpi.com/article/10.3390/jof10080547/s1, Section S1: Schematic overview of the volatile metabolite assay; Section S2: Chemical screening procedure.
