# Supplementary material for: Identification of Secondary Metabolites by UHPLC-ESI-HRMS/MS in Antifungal Strain Trichoderma harzianum (LBAT-53)
Source: J Fungi (Basel). 2024 Aug 3;10(8):547. doi: 10.3390/jof10080547 (PMC11355277; doi:10.3390/jof10080547)
Supplement: Supplementary file 1 [file jof-10-00547-s001.zip › jof-3064713-supplementary.pdf]

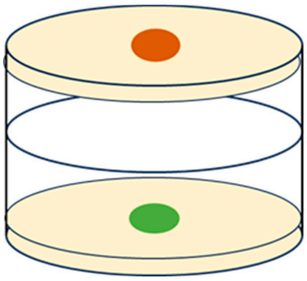

Section S1. Schematic overview of the volatile metabolite assay. Culture discs of both fungi (5 mm) were inoculated in the center of Petri dishes with PDA (90 mm) and the lids were removed. The bottoms containing antagonist and pathogen were placed together and sealed using Parafilm® and incubated at 30 ± 1 °C under dark conditions. The pathogen was in the upper plate in order to avoid any interference by antagonistic spores in the plate inoculated with Foc. As control, a bottom containing the pathogen was used overlapping with another containing only PDA medium.

Section S2. Chemical screening methodology.

Table S1. Reagent Preparation for chemical screening

Reagents	Preparation
Dragendroff's reagent	Stock solution: 5.2gm Bismuth carbonate + 4gm sodium iodide + 50mL glacial acetic acid, boiled for few min., After 12 hr. precipitated sodium acetate crystals are filtered by sintered glass funnel; 40mL filtrate + 160mL ethyl acetate + 1mL distilled water, (stored in amber-coloured glass bottle).<br>Working solution: 10mL stock solution + 20mL acetic acid + distilled water to make final volume 100mL
Hager's reagent	Saturated aqueous solution of picric acid
Mayer's reagent	Solution A : 1.358 mg mercuric chloride + 60mL distilled water<br>Solution B : 5 mg potassium iodide + 10mL distilled water<br>Working solution: solution A + solution B + distilled water to make final volume 100ml
Wagner's reagent	1.27mg iodine + 2mg potassium iodide + distilled water to make final volume 100mL
Fehling's solutions	Solution A: 34.66 gm copper sulphate + distilled water to make final volume 100mL.<br>Solution B: 173 mg potassium sodium tartarate + 50mg NaOH + distilled water to make 100 mL

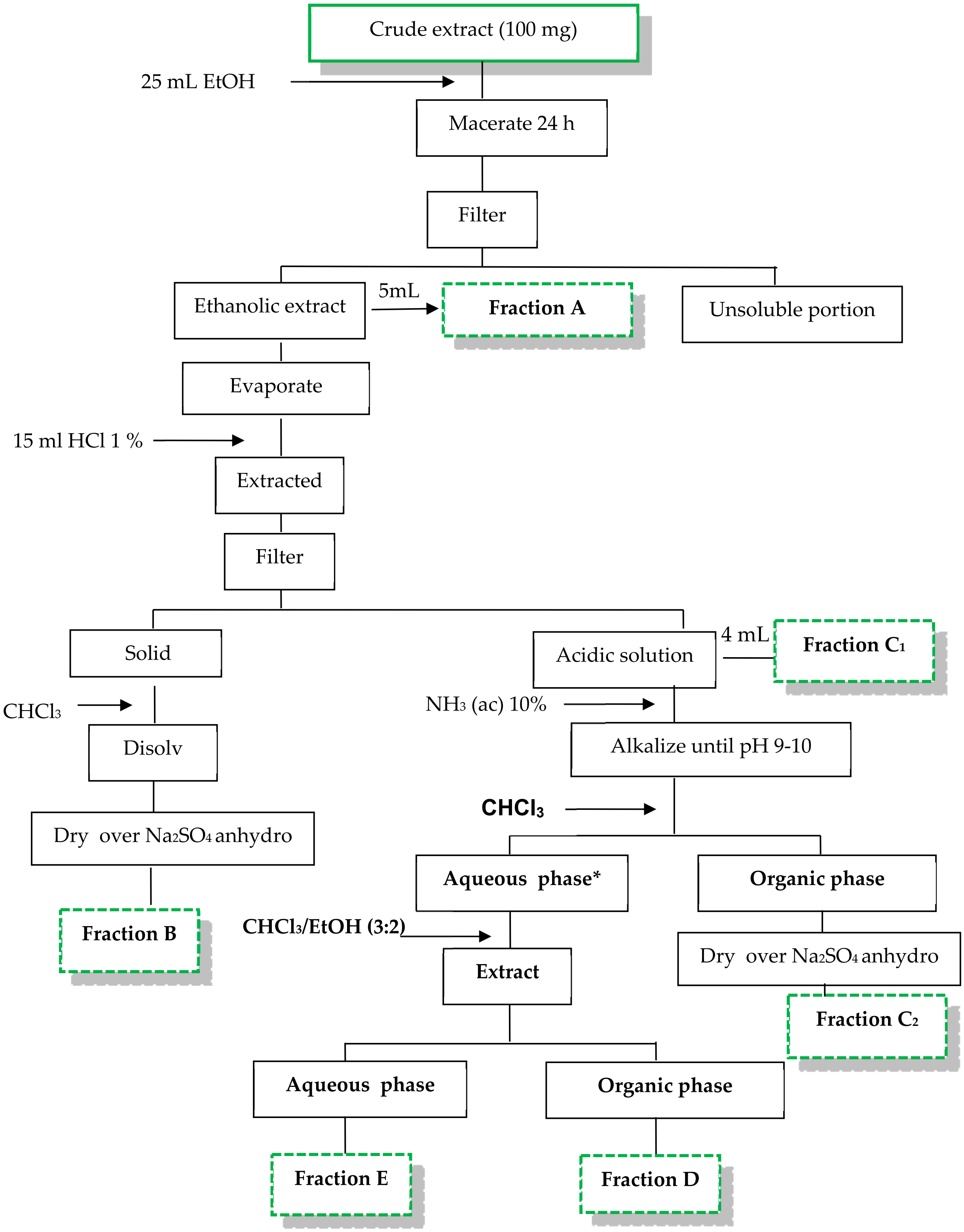

* Wash with a semisaturated solution of Na_2SO_4.

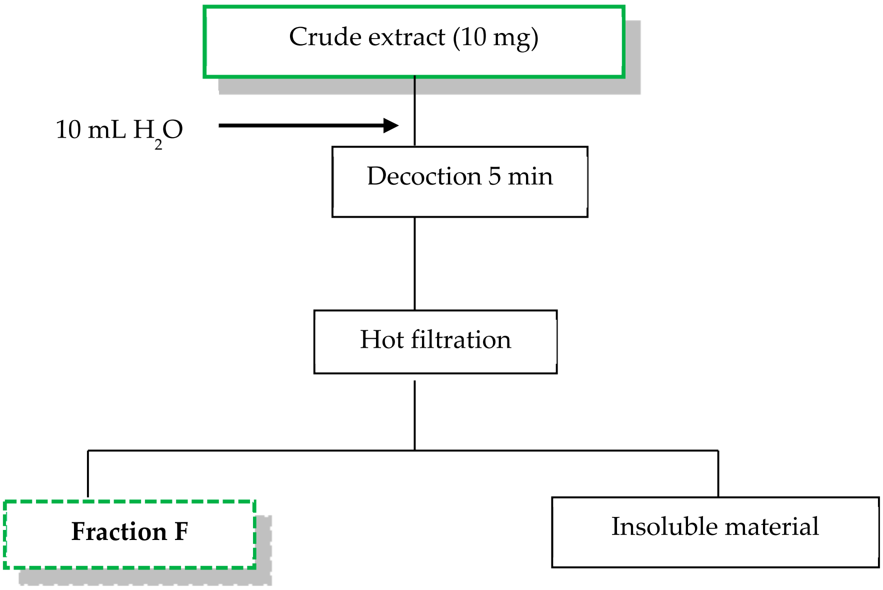

Figure S1. Workflow diagram.

Chemical screening methodology.

100 mg of crude extract is extracted with 25 mL of 95% ethanol by maceration, assisted with ultrasound for 24 hours. The alcoholic extract is filtered by gravity and the solid is discarded. From the ethanolic extract, 5 mL are separated, which constitutes fraction A. the rest of the extract is concentrated to dryness at reduced pressure and not more than 45 °C. The crude thus obtained is extracted with 15 mL of 1% HCl with slight heating (30-40 °C). The acid solution is filtered by gravity and the insoluble residue is extracted again with 5 mL of 1% HCl. It is filtered and the acid solutions are reunited. The insoluble portion is redissolved in 7 mL of chloroform, with slight heating and agitation, then a portion of anhydrous sodium sulfate is added to dry the chloroformic phase; it is filtered and the obtained solution is what constitutes fraction B. From the acid solution obtained previously 4 mL are separated that constitute the fraction C1. Subsequently, the rest of the acid solution is alkalinized with ammonia until pH = 9-10, then it is extracted in two successive operations with chloroform (25 mL each extraction), the organic phases are reunited and dried with anhydrous sodium sulfate, it is filtered and the obtained chloroform phase constitutes fraction C2. The aqueous phase is removed from the separatory funnel and semi-saturated with anhydrous sodium sulfate, then extracted with a $CHCl_3$/EtOH (3:2) mixture in two successive operations of 25 mL each. The organic phases are brought together and the aqueous phase is removed from the separatory funnel. The organic phase is placed again in the separatory funnel and is extracted with 10 mL of semi-saturated solution of anhydrous sodium sulfate, the organic phase is separated and the aqueous phase of the wash is reunited with the aqueous phase of the extraction with ($CHCl_3$/EtOH) (3:2), this union of aqueous phases constitutes the E fraction. The organic phase ($CHCl_3$/EtOH) (3:2) is dried with anhydrous sodium sulfate and filtered, the filtrate constitutes fraction D.

Additionally, 10 mL of distilled water is added to 10 mg of dry crude extract and a decoction is made for 5 minutes. It is filtered by gravity and hot, the aqueous extract thus obtained constitutes the fraction F.

On each of the fractions of increasing polarity obtained by the previous fractionation, the assays corresponding to the different groups of metabolites to be detected are performed.

<u>**Fraction A**</u>: Tests are performed to detect compounds with amino groups, tannins and phenolic compounds.

- **Amino groups**: To 0.5 mL of fraction A, three drops of 2% ninhydrin solution in ethanol were added. It was heated for 5 minutes in a boiling water bath and compared with the color produced with the original solution. It was considered positive when a purplish color developed.
- **Tannins and phenols**: 1 mL of fraction A was taken, evaporated to dryness and the residue was redissolved in 1 mL of physiological saline (0.9%). It was filtered, divided into two portions of 0.5 mL

To perform the tannin and phenol assays, with three drops of 0.5% gelatin solution and 1% iron (III) chloride, respectively. The formation of a precipitate with gelatin is indicative of the presence of tannins. Phenolic compounds give colorations ranging from blue or green to violet, brown or black, precipitate formation may occur.

<u>**Fraction B**</u>: Tests for triterpene-type compounds and/or steroids and quinones were performed.

- **Triterpenes/steroids:** (Liebermann-Bürchard reaction). To 1 mL of fraction B 1 mL of acetic anhydride was added and mixed in a test tube. Two or three drops of concentrated sulfuric acid were allowed to fall down the walls of the tube. The test is considered positive when a blue, green, orange, purple or violet color appears.
- **Quinones** (Börntrager reaction): 1 mL of fraction B was taken, evaporated to dryness and redissolved in 1 mL of toluene. 0.5 mL of 5% NaOH solution was added, stirred and the aqueous and organic phases were allowed to settle. The test is considered positive if the aqueous phase takes a red, orange or yellow color, also purplish; associated to the presence of naphthoquinones, anthraquinones, anthrones and/or anthranols.

<u>**Fraction C1**</u>: Preliminary tests for alkaloids (Mayer, Wagner, Hager and Dragendorff reagents) were performed. 1 mL of fraction C1 was treated with three drops of each of the reagents. The presence of turbidity or a white or yellow precipitate indicates a positive test for Mayer's reagent, brown precipitate (Wagner's reagent), yellow precipitate or crystals (Hager's reagent) and reddish-brown precipitate (Dragendorff's reagent).

<u>**Fraction C2**</u>: A confirmatory assay for alkaloids, triterpenes/steroids and cardenolides was performed. Four mL of the fraction was taken and concentrated to half volume, the rest of the fraction was evaporated to dryness and redissolved in 3 mL of 1% HCl and filtered.

- **Triterpenes/steroids**: on 1 mL of the concentrated aliquot, the test for triterpenes/steroids is performed in the same way as for fraction B.

- **Cardenolides:** a drop of the concentrate is deposited on a filter paper followed by a drop of the freshly prepared Kedde's reagent (equal parts of an aqueous solution of KOH 7% and 3,5-dinitrobenzoic acid at 2% in methanol), it is considered positive if a purple or violet coloration appears.
- **Alkaloids:** alkaloid detection assays similar to fraction C1 were performed on the acid solution.

Fraction D: Flavonoids, cardenolides, alkaloids, proanthocyanidins and triterpenes/steroids were tested. The fraction was brought to dryness and redissolved in 4 mL of ethanol and divided into rational portions to perform the assays.

- **Flavonoids** (Shinoda reaction): to a 0.2 mL portion 0.1 mL concentrated HCl was added. It was shaken and magnesium ribbon were added. It was shaken again and allowed to stand for 5 minutes. It was diluted with 2 mL of water and stirred with 0.4 mL of amyl alcohol. The phases were allowed to decant and the color of the amyl phase was observed. A positive assay is one that colors this phase bright yellow, orange, red, pink, purple, blue or brown. The result is compared with a blank in which the addition of the magnesium ribbon is omitted.
- **Cardenolides**: the assay was performed in a test tube on 0.2 mL of ethanolic solution to which 1 mL of Kedde's reagent was added, a blank was made by replacing the reagent with an equal amount of 0.5 mol/L KOH.
- **Alkaloids**: they were evaluated in the same way as described for fraction C1 with a drop of reagent, in a porcelain plate with perforations. Previously, ethanol was evaporated in a water bath and redissolved in 0.2 mL of 1% HCl.
- **Proanthocyanidins/ catechins** (Rosenheim reaction): equal volume of concentrated HCl was added to 0.4 mL of the ethanolic solution. It was heated in a water bath at 90 °C for 10 min, then 0.2 mL of water and 1 mL of amyl alcohol were added. A positive assay colors the alcohol red (proanthocyanidins) or brown (catechins), the coloration obtained is compared with a blank in which the addition of the acid. Magnesium is omitted.
- **Triterpenes/steroids**: another portion of the ethanolic solution (0.4 mL) was taken and evaporated to dryness in water bath. It was redissolved with 0.4 mL of chloroform and the assay was performed as described above for fraction B.

Fraction E: Flavonoids, proanthocyanidins/catechins and reducing sugars were tested. For both flavonoids and proanthocyanidins, the assay was performed on 2 mL in the same way as for **Fraction D**, in neither case requiring the addition of water.

For the detection of reducing sugars, 1 mL of the fraction was treated with 2 mL of a mixture in equal parts of Fehling's reagents A and B. It was heated in a water bath for 5-10 minutes. A positive assay corresponds to a green, orange or blue coloration with brick red precipitate.

Fraction F: Assays for saponins and amino groups were performed. 4 mL of solution F were placed in a test tube and shaken vigorously for 3 minutes, the appearance of abundant and stable foam during some minutes indicates the presence of saponins. For the detection of compounds with amino groups, the assay was carried out in the same way as for **Fraction A**.
